# Systematic Review and Meta-Analysis of Female Reproductive Health Following Ebola Virus Disease

**DOI:** 10.4269/ajtmh.23-0709

**Published:** 2024-12-17

**Authors:** Madison Drogy, Celia Glezer, Emily Engel, Nell Bond, Keith Pickett, Jeffrey Shaffer, John Schieffelin, Crystal Zheng

**Affiliations:** ^1^ School of Public Health and Tropical Medicine, Tulane University Newcomb–Tulane College, New Orleans, Louisiana;; ^2^ Department of Pediatrics, Section of Pediatric Infectious Diseases, School of Medicine, Tulane University, New Orleans, Louisiana;; ^3^ Department of Immunology and Microbiology, School of Medicine, Tulane University, New Orleans, Louisiana;; ^4^ Rudolph Matas Library of the Health Sciences, Tulane University, New Orleans, Louisiana;; ^5^ Department of Biostatistics and Data Science, School of Public Health and Tropical Medicine, Tulane University, New Orleans, Louisiana;; ^6^ Department of Medicine, Section of Infectious Diseases, School of Medicine, Tulane University, New Orleans, Louisiana

## Abstract

The viral hemorrhagic fevers Lassa fever (LF) and Ebola virus disease (EVD) have been documented to cause long-term health problems in survivors. Limited studies have noted the presence of adverse reproductive health outcomes, including menstrual irregularities and pregnancy loss, after recovery from infection. The objective of this systematic review and meta-analysis is to summarize existing knowledge surrounding reproductive health in female survivors of LF and EVD. Literature was gathered from PubMed, Embase, Ovid Medline, Cumulative Index of Nursing and Allied Health (CINAHL) Complete, Web of Science, and Global Health databases and subsequently reviewed in Covidence. Included studies described at least one reproductive health outcome in women after recovery from EVD or LF. Thirteen studies were identified in the systematic review, all of which only discussed reproductive health in EVD survivors. No studies of reproductive health among survivors of LF were identified. The included studies were conducted in Guinea, Sierra Leone, and Liberia, and they reported irregular menstruation, pregnancy loss, decreased libido, pelvic inflammatory disease, sexual dysfunction, female reproductive odor, and genital problems/infections among survivors. In a meta-analysis of nine studies, 14.0% of female EVD survivors experienced any adverse reproductive health outcome. However, there was significant heterogeneity among the included studies. This study highlights the health problems faced by female EVD survivors and underscores the need for more research surrounding the effects of viral hemorrhagic fevers on women’s health.

## INTRODUCTION

Ebola virus disease (EVD) and Lassa fever (LF) are viral hemorrhagic fevers (VHFs) with both acute and long-term impacts. Postacute disease sequelae of EVD and LF have been well described among survivors, but descriptions of female reproductive health among this population have been limited. Long-term health problems in LF survivors, including hearing loss and ocular complications, have been described in studies conducted by Li et al.,^1^ Cummins et al.,^2^ and Mateer et al.^3^ Post-Ebola syndrome has been described by several studies conducted in West Africa, including the Partnership for Research of Ebola Virus (PREVAIL III), which found that symptoms, including urinary frequency, headache, fatigue, muscle pain, memory loss, and joint pain, were reported significantly more often in Liberian EVD survivors compared with uninfected contacts. PREVAIL III also reported higher prevalences of amenorrhea, decreased libido, and reproductive odor among female EVD survivors, but these impacts to women’s health were not a focus of the study.^4^ In the Liberian Ebola Survivors Cohort, Wohl et al.^5^ reported that 75% of survivors experienced at least one symptom, with 86% of these symptoms highly interfering with life. In a study conducted by Bond et al.,^6^ Sierra Leonean EVD survivors had fever, headache, joint pain, muscle pain, numbness/tingling, and difficulty sleeping at significantly higher levels than uninfected contacts. Similar symptoms were found in the PostEboGui study conducted by Diallo et al. in Guinea.^7^ In addition to physical symptoms, EVD has negative impacts on survivors’ mental health and quality of life.^8^^,^^9^ In studies with longitudinal follow-up, the prevalence of almost all post-Ebola syndrome symptoms decreased over time.^4^^,^^5^^,^^7^

Few studies have investigated the specific female reproductive health impacts experienced by survivors of EVD and LF, although adverse reproductive health outcomes, such as fetal loss, are well documented during acute illness.^10^^,^^11^ In addition, several studies have noted higher mortality rates among pregnant women with LF and EVD.^12^^–^^14^ More comprehensive research is needed to determine the extent of reproductive health impacts among LF and EVD survivors after recovery from acute illness as they are associated with cardiovascular disease, osteoporosis, infertility, and decreased quality of life.^15^ For example, abnormal menstruation is associated with worse overall health status, health-related anxiety, and decreased productivity, whereas decreased libido is associated with low self-esteem, anxiety, depression, and relationship difficulties.^16^^–^^19^ Globally, the prevalence of amenorrhea ranges from 3% to 4%, whereas a systematic review found the prevalence to be 5–12% in developing countries, indicating the potential role of socioeconomic status and resource availability.^20^^–^^22^ A multicenter prospective cohort study in sub-Saharan Africa (SSA) reported the prevalences of miscarriages (1.6%), stillbirths (1.7%), and neonatal mortality (2.0%) in this region.^23^ Similarly, a cross-sectional survey of the Demographic and Health Surveys in 10 SSA countries found a 1.2% prevalence of stillbirths, and a large global review study found a 3.2% prevalence of stillbirths in SSA.^24^^,^^25^ Moreover, in West Africa, the prevalence of female genital mutilation, which is associated with decreased libido and sexual dysfunction, ranges from 70% to 90%.^26^ Studies among women in Nigeria have demonstrated high prevalences of sexual dysfunction from 53% to 63%.^26^^–^^28^ Thus, although this present study is novel in its breadth of research discussing reproductive health after EVD survival, it is important to contextualize the findings within the baseline prevalence of adverse female reproductive outcomes in the larger population.

This systematic review and meta-analysis aims to identify published studies with information regarding adverse women’s health outcomes after VHF and be the first to consolidate these findings. A greater understanding of this topic will allow for more effective, evidence-based solutions to the health problems that female VHF survivors face.

## MATERIALS AND METHODS

### Systematic review.

A systematic review was performed according to the established Preferred Reporting Items for Systematic Reviews and Meta-Analyses guidelines. This systematic review was conducted from October 2021 through November 2023. The initial literature search was conducted on November 19, 2021, and the most recent search was conducted on November 30, 2023. There was no publication date cutoff included in the literature search. All English language primary sources, publications, and geographic locations were considered. Literature was gathered from PubMed, Embase, Ovid Medline, Cumulative Index of Nursing and Allied Health (CINAHL) Complete, Web of Science, and Global Health databases using the following key search terms: Ebola, survivor, Lassa fever, hemorrhagic fever, and convalescence (Supplemental Table 1). Articles were uploaded into Covidence (Veritas Health Innovation, Melbourne, Australia), and duplicates were removed. Abstracts, tables, and figures were reviewed for each study to determine if inclusion criteria were met. Included studies were required to be primary sources reporting at least one reproductive health outcome affecting women (e.g., menstrual irregularities, pregnancy loss, infertility, dyspareunia, or decreased libido). These outcomes needed to be reported in LF or EVD survivors. Any studies reporting outcomes exclusively during the acute stage of illness were excluded. A full-text review was then conducted to confirm inclusion in the systematic review. Two reviewers (M. Drogy and C. Glezer) read and evaluated each study. Any discordances were settled by a third senior reviewer (C. Zheng).

Data extracted from each study included the following: country of study, sample size, average age of female participants, average time from Ebola treatment unit (ETU) discharge to first visit, reproductive health outcome(s) described, disease (EVD or LF), prevalence of women experiencing the specified reproductive health outcome, and key findings. Sample size was defined as the number of study participants who responded to each outcome evaluated.

### Meta-analysis.

The frequencies, percentages, and standard errors (SEs) for each reproductive health outcome investigated were extracted. Response proportions were considered the proportion of study participants who reported experiencing the outcome of interest. CIs and SEs were calculated based on a single normally distributed proportion $\left(SE=\;\sqrt{\frac{p\left(1-p\right)}{n}}\right)$, where *p* and *n* represent the response proportions and sample sizes, respectively. Sample sizes (*n*) were considered the number of study participants who gave a response to each question investigated. If a study analyzed multiple subgroups separately, *n* was defined based on the number of participants who gave a response for the outcome of interest in each subgroup. CIs were based on a standard normal distribution with a 5% type I error rate and calculated as 1.96 times the SE for each reported proportion. Studies that did not provide a response proportion among only female participants were excluded.

The Stata Meta-Analysis Workflow and Metaprop command (v. 16, StataCorp, College Station, TX) were used to generate forest plots.^29^ A random-effects approach was used to account for between-study heterogeneity. First, an overall analysis was performed to compare all included studies. This analysis provided a pooled response proportion, 95% CI, and overall *P*-value for heterogeneity. Next, the studies were stratified into three subgroups based on the reproductive health outcomes investigated: decreased libido, menstrual irregularities, and pregnancy loss. Studies that investigated multiple reproductive health outcomes could be included in more than one subgroup. The sample size (*n*) used in the pooled analysis was the sum of the number of responses given for each subgroup. The subgroup analysis provided a pooled response proportion, 95% CI, and *P*-value of heterogeneity for each outcome. The weights for the overall and subgroup meta-analyses were defined as their SEs. The type I error threshold was set at 5% in all hypothesis tests.

A post hoc power analysis was conducted in RStudio based on the ability to detect the overall estimate to within 5% points of its true value (the pooled-estimate approach) and the ability to detect between-study heterogeneity (the heterogeneity approach).^30^ The total number of effect sizes and the total sample sizes identified in the included articles were used for these calculations. For the pooled-estimate approach, we observed 76% power to estimate the proportion to within 5% points of its true value using a Cohen *h* value of 0.14. For the heterogeneity approach, Cohen *h* values for small (0.20) and medium (0.50) effect sizes were selected for the overall and subgroup analyses, respectively.^31^ We observed 97% and >75% power to detect between-study heterogeneity for the overall and subgroup analyses, respectively (Supplemental Table 2).

### Quality assessment.

Two methodologies were used to assess quality and bias by two independent reviewers (M. Drogy and C. Glezer) in Covidence. The first scoring system used an A, B, or C ranking. Cohort studies (prospective or retrospective) that included an uninfected comparison group or studies with sample size greater than 100 were given an “A.” Studies with sample size less than 100 were given a “B.” Case reports or case series were given a “C.” Article screening, quality assessment, and data extraction were performed in Covidence. The second scoring system assessed quality based on the NIH’s National Heart, Lung, and Blood Institute (NHLBI) scales (NHLBI case report scale or observational study scale as appropriate).^32^ Quality assessment was scored as “yes,” “no,” “not applicable,” or “no information” based on fulfillment of each scale domain. Graphing of publication quality results was conducted using the robvis tool.^33^

## RESULTS

### Systematic review.

**Figure 1.**
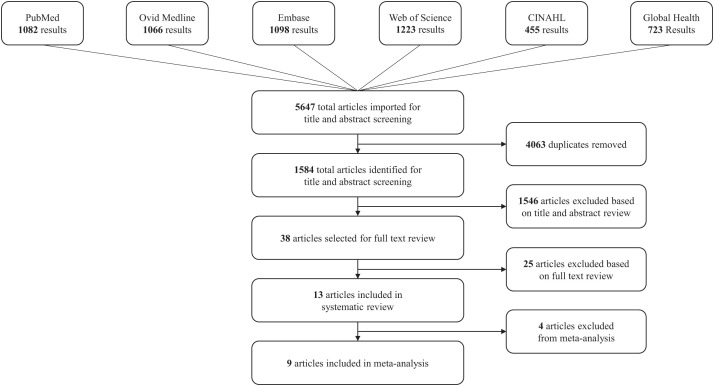
Systematic review literature search results selection process for included studies. A total of 13 studies were selected for inclusion in the systematic review, and 9 studies were selected for inclusion in the meta-analysis. CINAHL = Cumulative Index of Nursing and Allied Health.

A total of 1,584 studies were identified during the initial search after removing 4,063 duplicate studies that were found in two or more databases (Figure 1). Thirty-eight studies were selected for full-text review after excluding 1,546 articles based on title and abstract review. Among articles selected for full-text review, 23 were excluded because reproductive health outcomes were not investigated, and 2 were excluded because the study population included only acutely ill patients and not survivors. After excluding articles that did not meet the inclusion criteria, 13 studies detailing reproductive health outcomes after EVD in women were included in the systematic review (Table 1).^34^^–^^45^ There were no identified articles studying LF.

**Table 1:** Systematic review findings

Reference	Publication Year, Country, and Setting	Study Design	Female Sample Size	Age (years)*	Time from ETU Discharge to First Visit*	Reproductive Health Outcome^†^	Prevalence (%)	Quality Assessment^‡^	Additional Findings
de St. Maurice et al.^35^	2018, Liberia, EVD survivor clinic	Retrospective cohort study^§^	189	Mean 33 (range: 0.9–75)	Mean 288 days (range: 4–909)	Irregular menses, decreased libido, pelvic inflammatory disease	12.7%, 9.0%, 10.6%	A, 8	Survivors with depression were more likely to report decreased libido than those without depression. The proportion of visits for decreased libido decreased significantly over time. There was no significant difference in the prevalence of nonreproductive health symptoms between males and females.
Fallah et al.^38^	2016, Liberia, EVD survivor pregnancy clinics	Retrospective cohort study	70	Mean 26.4 (SD 5.7) among women with normal pregnancies, mean 28.8 (SD 6.8) among women with failed pregnancies	Range: 1–15 months	Miscarriage, stillbirth	22.1%,^ǁ^ 5.9%^ǁ^	B, 8	Of the 6 pregnancies that occurred within 2 months of ETU discharge, 50% resulted in stillbirths.
Godwin et al.^34^	2019, Liberia, EVD survivor clinic	Cross-sectional questionnaire	111	Mean 31.6 (SD 7.3)	Mean 709 days (SD 45)	Menstrual irregularities, spontaneous abortion, stillbirth	29.0%,^ǁ^ 47.8%,^ǁ^ 8.7%^ǁ^	A, 8	Mean time between ETU discharge and conception was not significantly different for women with successful pregnancies (220 days) vs. failed pregnancies (287 days).
Kamali et al.^41^	2016, United States and West Africa, hospital	Case report	1	29	154 days	Successful pregnancy	100%^ǁ^	C, 5	This is a case of a successful pregnancy in a woman who became pregnant 22 weeks after her disease resolution.
Mattia et al.^40^	2015, Sierra Leone, EVD survivor clinic	Cross-sectional, chart review	163	Median 29 (IQR: 20–36)	Median 121 days (range: 82–151)	Miscarriage among women with uveitis, ocular symptoms, auditory symptoms, and arthralgias	5.7%, 6.7%, 7.5%, 6.5%	A, 8	There was no significant difference in prevalence of miscarriages among survivors with uveitis, ocular symptoms, auditory symptoms, and/or arthralgias compared with those who did not report these symptoms.
Mohammed et al.^36^	2017, Sierra Leone, EVD survivor clinic	Retrospective cohort study^§^	423	Mean 28 (range: 0.25–70)	Median 114 days (range: 4–395)	Amenorrhea	21.4%^ǁ^	A, 9	Frequency of amenorrhea decreased over time.
Nanyonga et al.^45^	2016, Sierra Leone, community survey	Cross-sectional questionnaire	51	Median 29 (range: 10–74)	Not reported	Amenorrhea	2.5%	B, 5	–
Qureshi et al.^42^	2015, Guinea, survivors discharged from ETU	Cross-sectional questionnaire	105	Mean 38.9 (SD 11.9)	Mean 103.5 days (SD 47.9)	Deceased libido, sexual dysfunction	23.1% (includes both sexes), 20.0% (includes both sexes)	A, 11	There was no significant difference in prevalence between the acute convalescent group (0–90 days since discharge) and the subacute convalescent group (90–210 days since discharge).
PREVAIL III^4^	2019, Liberia, community survey	Prospective cohort^§^	528	Survivors: median 29 (IQR: 19–40), uninfected contacts: median 23 (IQR: 13–34)	Median 358 days (IQR: 313–405)	Amenorrhea, decreased libido, female reproductive odor	14.2% (baseline), 12.6% (12 months), 17.6% (baseline), 10.7% (6 months), 5.7% (baseline)	A, 12	EVD survivors were more likely than uninfected contacts to report amenorrhea (OR: 1.76), decreased libido (OR: 4.34), and female reproductive odor (OR: 2.46). At 6 and 12 months, the prevalences of these symptoms declined but were still higher in survivors compared with uninfected contacts.
Tiffany et al.^43^	2016, Sierra Leone, EVD survivor clinic	Longitudinal chart review^§^	74	Mean 24.7 (SD 12.7)	Mean 51.1 days (SD 41.2)	Amenorrhea, genital/urinary tract infection/STI	10.7%,^ǁ^ 22.8% (includes both sexes)	B, 9	–
Guetiya Wadoum et al.^39^	2017, Sierra Leone, EVD survivor clinic	Longitudinal questionnaire^§^	148	Mean 27	Range: 9–26 months	Oligomenorrhea or premature menopause, miscarriage, loss of sexual desire	8.8%, 10.8%, 4.7%	A, 8	–
Wilson et al.^37^	2018, Liberia, community survey	Cross-sectional questionnaire	174	Median 30 (range: 18–70)	Range: 0–12 months	Menstrual problems	19.7%	A, 9	91% of EVD survivors who reported menstrual irregularities experienced onset within the first 3 months after ETU discharge; 25% had resolution within 3 months, whereas 53% experienced persistent symptoms for 10–12 months.
Wing et al.^44^	2018, Sierra Leone, EVD survivor clinic	Retrospective cohort study^‡^	78	Median 21 (IQR: 14–30)	Median 109 days (IQR: 91–120)	Amenorrhea, genital problems	18.2%, 17.8% (includes both sexes)	B, 10	–

ETU = Ebola treatment unit; EVD = Ebola virus disease; IQR = interquartile range; OR = odds ratio; STI = sexually transmitted infection.

* Based upon the entire study population, including males if the study included males.

^†^ Reproductive health outcome as named in each study.

^‡^ Quality assessment scores are given in the form of a letter grade (A–C) and a score based on the National Heart, Lung, and Blood Institute scale. All National Heart, Lung, and Blood Institute scores are out of 14 except for the score for Kamali et al.,^41^ which is scored out of 9 based on the case report scale.

^§^ Includes multiple visits per patient.

^ǁ^ Among reproductive age women only.

The selected studies included five cross-sectional studies, four retrospective cohort studies, two longitudinal studies, one prospective cohort study, and one case report. These studies described reproductive health outcomes, including menstrual irregularities (*n* = 9), pregnancy loss (*n* = 5), decreased libido/sexual dysfunction (*n* = 4), pelvic inflammatory disease (*n* = 1), female reproductive odor (*n* = 1), and genital/urinary tract infection/sexually transmitted infections (STIs; *n* = 1). This review also extracted data when available regarding the onset and persistence of adverse reproductive health outcomes over time. The median time from ETU discharge to first visit was 121 days (calculated as the overall median of the mean or median time since ETU discharge provided in individual studies), and the overall range was 1–26 months (calculated using the outer limits of the ranges provided in individual studies). Except for Godwin et al.,^34^ the mean or median time to first visit in all other studies was within the first year after ETU discharge. The mean time between ETU discharge and measurement of the pregnancy outcome in Godwin et al.^34^ was 709 days, but the mean number of days between ETU discharge and conception was 220 and 287 days for live birth and failed pregnancy, respectively.

The PREVAIL III study in Liberia was the only prospective longitudinal study; it had the largest sample size, and it was the only study with an uninfected control group. The study followed participants for 1 year at 6-month intervals, and it included 528, 467, and 458 female EVD survivors at the enrollment, 6-month, and 12-month visits, respectively.^4^ Participants presented for their enrollment visit a median of 358 days post-ETU discharge. Investigators tracked self-reported symptoms, including amenorrhea, female reproductive odor, and decreased libido, as well as nonreproductive symptoms. The largest study to focus specifically on reproductive health was a cross-sectional study conducted by Godwin et al.^34^ among 111 women ages 18–45 years who enrolled in the Longitudinal Liberian Ebola Survivor study. Study participants were asked about their menstrual and pregnancy history in a private interview setting.^34^

#### Irregular menses.

Nine studies reported data on “menstrual irregularities,” “amenorrhea,” and “oligomenorrhea.” All nine studies found that female EVD survivors reported increased prevalences of menstrual irregularities ranging from 2.5% to 29.0% after disease recovery compared with before disease onset. PREVAIL III reported odds ratios of 1.76 (95% CI: 1.27–2.43) and 1.60 (95% CI: 1.13–2.27) for amenorrhea between survivors and uninfected contacts at the enrollment visit and the 12-month visit, respectively. PREVAIL III also reported the prevalences at enrollment among female EVD survivors of painful periods (20.5%), menopause (10.6%), abnormal vaginal bleeding (1.3%), menarche (0.2%), and postmenopausal symptoms (1.1%), although these were not significantly different compared with uninfected contacts. The study by Godwin et al.^34^ was the only study that provided a more granular description of menstrual irregularities. This study found that of the 27 women who experienced new menstrual irregularities post-EVD, 48.1% were classified as having oligomenorrhea (“infrequent menstruation, reduced duration of flow, and light or scanty flow”), 18.5% experienced amenorrhea (“absence of menstruation for at least 3 months”), and 14.8% experienced dysmenorrhea (“abdominal pain, cramping, or back ache during menstruation”) or menorrhagia (“frequent menstruation, increased duration of flow, and/or heavy flow”).

Data regarding the prevalence of irregular menses based upon the time since ETU discharge were also extracted when available. de St Maurice et al.^35^ found that the prevalence of menstrual irregularities did not change over a 30-month period post-ETU discharge.^36^ However, Mohammed et al.^36^ reported a decrease in the frequency of amenorrhea over time. The overall prevalence of amenorrhea at any visit was 21.4%, but when broken down by time interval since ETU discharge, the prevalences were 11.3% (4–144 days), 11.6% (145–254 days), 4.0% (255–358 days), and 3.7% (359–504 days).^36^ Similarly, PREVAIL III found a reduction in the prevalence of amenorrhea from 14.2% at the baseline visit to 12.6% at the 12-month visit. However, the prevalence remained significantly higher in survivors compared with uninfected contacts at both time points. Wilson et al.^37^ found that of the 19.7% of female survivors with menstrual irregularities, 91% experienced onset within the first 3 months post-ETU discharge. Moreover, 25% of the female survivors with menstrual irregularities resumed normal menses within 3 months, whereas 53% had persistent symptoms for 10–12 months.^37^

#### Pregnancy loss.

Five studies discussed pregnancy outcomes in EVD survivors, including four observational studies that identified “adverse pregnancy outcomes” or “pregnancy loss” and one case report of a successful pregnancy. Fallah et al.^38^ conducted a review of 70 EVD survivors who sought care for pregnancy at designated hospitals in Liberia, finding that 27.9% of participants experienced either a stillbirth (5.9%) or miscarriage (22.1%) after disease recovery. Of the six pregnancies that occurred within 2 months of ETU discharge, 50% resulted in a stillbirth.^38^ Similarly, Godwin et al.^34^ reported that of the 29 EVD survivors who had become pregnant after disease recovery, 47.8% experienced a spontaneous abortion, and 8.7% experienced a stillbirth. There was no significant difference in the time between ETU discharge and conception among individuals whose pregnancies ended in a live birth (220 days) versus pregnancy loss (287 days). The study by Guetiya Wadoum et al.,^39^ conducted at a mobile EVD survivor health clinic in Sierra Leone, found that 10.8% of all women presenting for general care reported a pregnancy loss after recovery. In a Sierra Leonean survivor clinic, Mattia et al.^40^ reported prevalences of miscarriage between 5.7% and 7.5% among EVD survivors who experienced concomitant uveitis, ocular problems, auditory problems, and arthralgias; there were no significant differences in the prevalences of miscarriage between these groups. Of note, Kamali et al.^41^ discussed a case of a successful pregnancy after EVD in a woman from West Africa who received prenatal care in the United States.

#### Decreased libido.

Four studies reported decreased libido and sexual dysfunction as post-Ebola syndrome outcomes. de St Maurice et al.^35^ found that 8.9% of female survivors who presented to an EVD survivor clinic in Liberia reported decreased libido, which was also found to be associated with depression. The proportion of visits for decreased libido decreased significantly from 0 to 30 months after ETU discharge. PREVAIL III found a reduction in female decreased libido from 17.6% at the enrollment visit to 10.7% at the 6-month visit, although female EVD survivors were more likely to report decreased libido compared with uninfected contacts at both visits, with odds ratios of 4.34 (95% CI: 3.0–6.29) at enrollment and 6.89 (95% CI: 3.91–12.11) at 6 months. Qureshi et al.^42^ surveyed 105 EVD survivors (71 men and 34 women) discharged from a large ETU in Guinea and found that 23.1% of survivors experienced decreased libido and that 20.0% experienced sexual dysfunction. These outcomes were found at similar frequencies during the acute convalescent (0–90 days postdischarge) and subacute convalescent (90–210 days postdischarge) phases. However, sex-stratified results were not available in the manuscript.^42^

### Meta-analysis.

**Figure 2.**
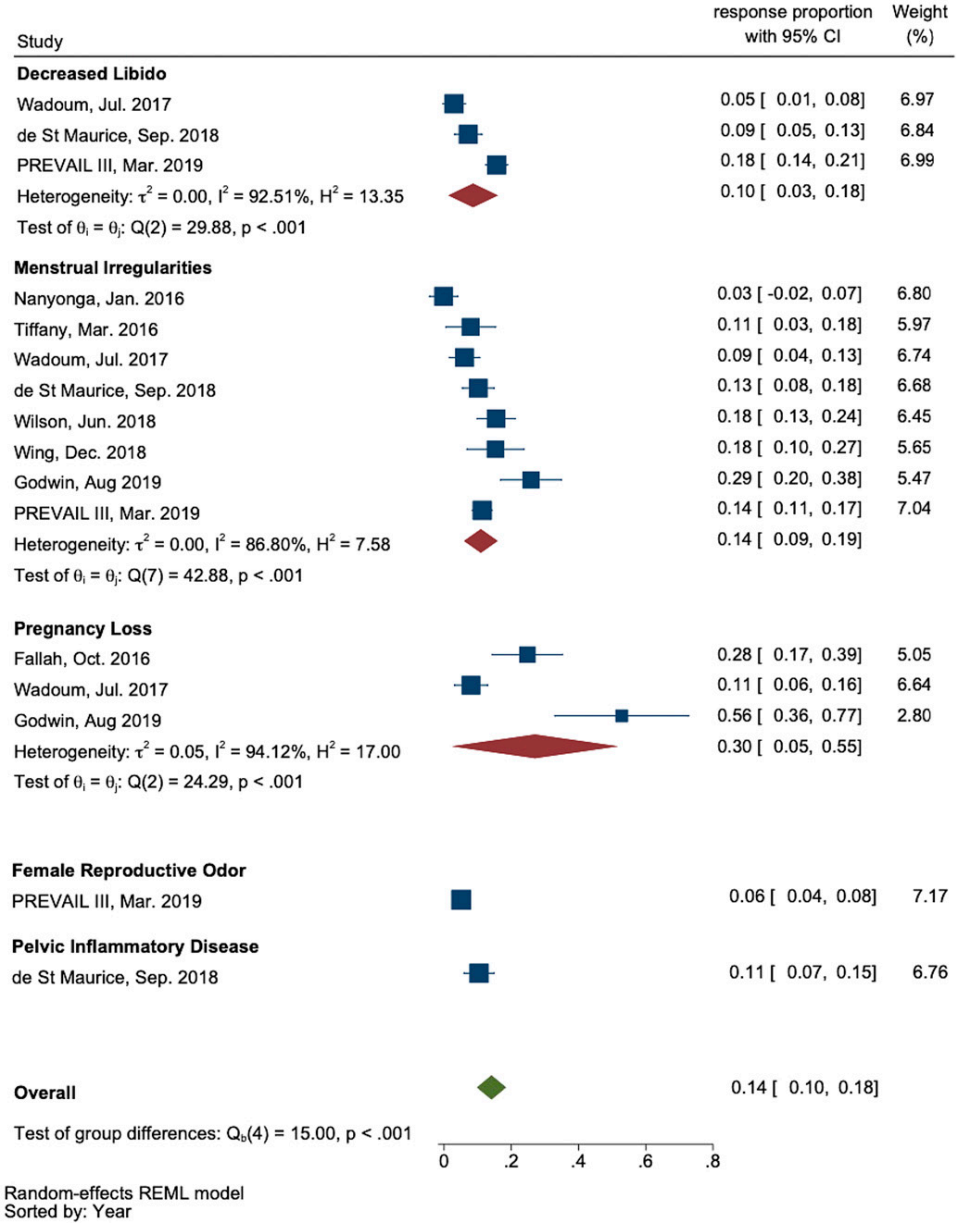
Meta-analysis forest plot of response proportions for reproductive health outcomes. Square represents individual study response proportion. Diamond represents pooled response for each group. PREVAIL III = Partnership for Research of Ebola Virus; REML = restricted maximum likelihood.

Nine of the 13 studies identified in the systematic review were included in the meta-analysis (Figure 2). The study by Kamali et al.^41^ was excluded because it described a single case study and did not include a response proportion. The study by Mattia et al.^40^ was also excluded because it only reported response proportions for menstrual irregularities stratified across other symptoms and thus, did not report an overall frequency. The study by Qureshi et al.^42^ was excluded because the response proportions were not stratified by sex. Further, the study by Mohammed et al.^36^ was excluded because the study only reported the frequency of amenorrhea using total visits rather than individuals as the denominator. Certain outcomes reported in Tiffany et al.^43^ (“genital/urinary tract infection/STI”) and Wing et al.^44^ (“genital problems”) were not stratified by sex and were excluded from the overall pooled proportion analysis. However, the prevalences of “menstrual irregularities” reported in these studies were still included in the meta-analysis because response proportions were reported specifically for females.

The pooled proportion of women who experienced any adverse reproductive health outcome across the included nine studies was 0.14 (95% CI: 0.10–0.18), with significant heterogeneity (*P* <0.001). Subgroups analyzing decreased libido, menstrual irregularities, and pregnancy loss included three, eight, and three studies, respectively. Pooled response proportions were calculated for decreased libido (0.10, 95% CI: 0.03–0.18), menstrual irregularities (0.14, 95% CI: 0.09–0.19), and pregnancy loss (0.30, 95% CI: 0.05–0.55). Each subgroup demonstrated high heterogeneity.

### Publication quality assessment.

**Figure 3.**
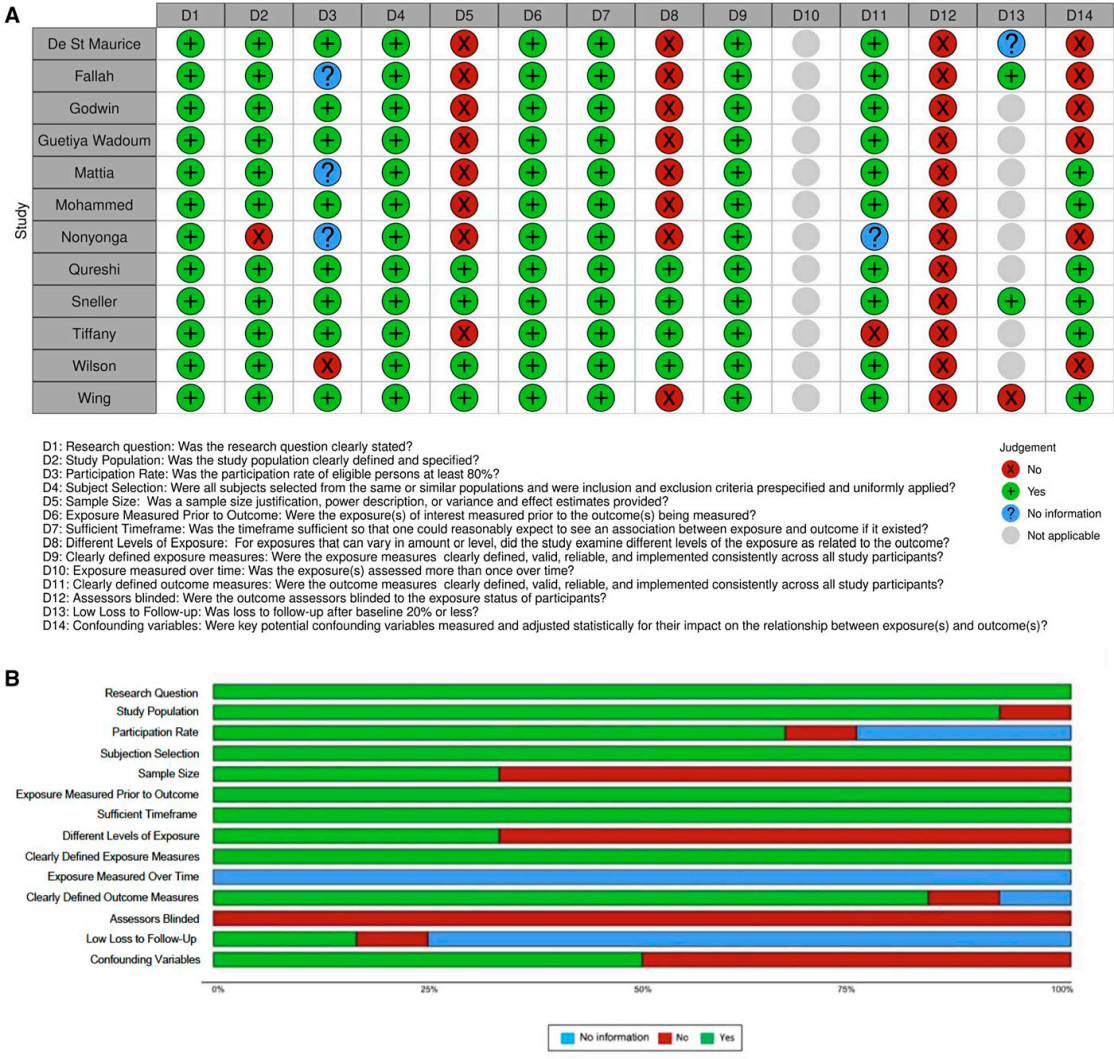
Quality assessment for the included studies. (**A**) The quality assessment summary figure. (**B**) The proportion of studies meeting each domain of the National Heart, Lung, and Blood Institute scale.

Eight studies with either an uninfected comparison group or a sample size greater than 100 were rated “A,” four studies with sample size less than 100 were rated “B,” and one case report was rated “C” (Table 1). On the NHLBI observational study scale, all studies included a clear research question, conducted subject selection from similar populations, measured exposure before outcome, had a sufficient time frame, and defined valid and reliable outcome measures. Assessors were not blinded to the exposure status of participants in any study (Figure 3; Supplemental Table 3). Notably, Qureshi et al.^42^ and PREVAIL III were rated “A” and fulfilled 11 of 14 and 12 of 14 NHLBI assessment criteria, respectively. The NHLBI scale for assessing case reports was used to evaluate Kamali et al.^41^ (Supplemental Table 4).

## DISCUSSION

The adverse female reproductive health outcomes reported after EVD recovery pose significant impacts on the overall health and well-being of female EVD survivors. This study is the first to conduct a systematic review on the topic, and it identified 13 studies that examined the association between EVD and reproductive health outcomes in women. The most reported outcomes were menstrual irregularities, pregnancy loss, and decreased libido. Additional symptoms reported in at least one study were infections/pelvic inflammatory disease, female reproductive odor, sexual dysfunction, and genital problems. In a pooled meta-analysis of data extracted from these studies, the overall prevalence of female EVD survivors who experienced any adverse reproductive health outcome was 14.0%.

The most investigated reproductive health outcome was menstrual irregularities. The pooled prevalence of menstrual irregularities was 14.0%, ranging from 2.5% to 29.0%. The wide range of prevalences may be explained in part because of the varied definition of menstrual irregularities in each study; some broadly used the term “menstrual irregularities,” whereas others used more specific terms, such as “amenorrhea,” “premature menopause,” or “oligomenorrhea.” Additionally, the variations in the prevalence calculations used in each study contributed substantially to the range of reported prevalences. For example, the denominator used to calculate prevalence in Nanyonga et al.^45^ was all female participants who ranged in age from 10 to 74 years and included women for whom this outcome would not be applicable, thus likely leading to an underestimated prevalence of amenorrhea (2.5%). On the other hand, the inclusion of nonreproductive age women could potentially lead to an overestimated prevalence of menstrual irregularities because of symptoms associated with menopause. When limiting to studies that calculated prevalence using women of reproductive age only, the range of menstrual irregularities was 10.7–29.0%. The pooled prevalence of menstrual irregularities among EVD survivors observed in this meta-analysis (14.0%) was higher than those reported among uninfected contacts in PREVAIL III (10.2%) and in previous studies conducted in similar settings (5.0–12.0%),^22^ indicating that EVD may uniquely contribute to irregular menstruation.

The prevalence of pregnancy loss in female EVD survivors ranged from 10.8% to 56.5%, with a pooled prevalence of 30.0%. These studies variably defined outcomes in this category as miscarriage, stillbirth, spontaneous abortion, or pregnancy loss. The variation in the calculations of prevalence also considerably affected the observed prevalence. For example, Guetiya Wadoum et al.^39^ and Mattia et al.^40^ reported prevalences of pregnancy loss to be 10.8% and 5.7–7.5%, respectively, but these prevalences are likely an underestimate because the denominator was composed of all women, including women who never experienced pregnancy post-EVD. In contrast, Godwin et al.^34^ and Fallah et al.^38^ restricted the calculation of prevalence to only women who became pregnant post-EVD and reported higher prevalences of 28.0% and 56.5%, respectively. Previous studies among the general population in SSA have reported prevalence of stillbirth ranging from 1.2% to 3.2% and prevalence of miscarriage of 1.6%.^23^^–^^25^ In comparison, the prevalences of stillbirth (5.9%^38^ and 8.7%^34^, respectively) and miscarriage (22.1%^38^ and 47.8%^34^, respectively) among EVD survivors found in this systematic review were substantially higher. Although the prevalence of pregnancy loss is concerning, it is important to note that successful pregnancies post-EVD were documented. Kamali et al.^41^ described a successful pregnancy in an EVD survivor who conceived in West Africa but who received prenatal care and delivered her infant in the United States, highlighting the role that adequate medical care and resources play in mitigating adverse pregnancy outcomes.

The prevalence of decreased libido among female EVD survivors ranged from 4.7% to 17.6%, with a pooled prevalence of 10%. Data comparing the prevalence of decreased libido among female and male EVD survivors were conflicting. Guetiya Wadoum et al.^39^ reported a lower prevalence of decreased libido in females (4.7%) than males (10.2%), although these were not statistically compared, whereas de St Maurice et al.^35^ did not find a significant difference based on sex (9.0% in females and 11.8% in males). In PREVAIL III, the odds ratio comparing EVD survivors with uninfected contacts was higher for female decreased libido (4.34) than for male decreased libido/impotence (2.76). Further studies are needed to determine how the prevalence of decreased libido differs in EVD survivors by sex. It is important to consider that the prevalence of female genital mutilation ranges from 70% to 90% in West Africa, which could confound the interpretation of the contribution of EVD to the prevalence of decreased female libido.^26^ However, PREVAIL III found significantly higher rates of decreased libido in female EVD survivors compared with uninfected contacts who can be assumed to have similar rates of female genital mutilation. The prevalences of decreased libido reported by studies in this review, even in women with no history of EVD, are much lower than the prevalences of sexual dysfunction reported in other studies among women in neighboring Nigeria. This discrepancy may be explained by differences in terminology. For example, in a survey of women at a teaching hospital in Nigeria, 63% reported sexual dysfunction, although only 8.3% of those were because of “disorder of desire.”^26^^–^^28^

Several studies described other reproductive health outcomes experienced by female EVD survivors, including sexual dysfunction, genital problems, pelvic inflammatory disease, and genital/urinary tract/STIs. PREVAIL III also found a higher prevalence of female reproductive odor among EVD survivors than uninfected contacts. These findings suggest that EVD may disrupt normal vaginal flora or predispose to vaginal infections. Notably, there were no studies that investigated infertility in female EVD survivors. It has been documented that other viral illnesses, such as coronavirus disease 2019, can contribute to downstream infertility through various mechanisms, including inflammatory cytokines, reduction in ovarian reserve, and viral suppression of the hypothalamic–pituitary–ovarian axis.^46^ Given the prevalence of menstrual irregularities among EVD survivors, it could be hypothesized that infertility is an additional outcome of EVD survival that should be further investigated.

There were conflicting data regarding the persistence of all reproductive health outcomes over time in EVD survivors. Although de St Maurice et al.^35^ found that the prevalence of irregular menses did not change from 0 to 30 months post-ETU discharge, Mohammed et al.^36^ and PREVAIL III reported decreasing frequency of amenorrhea longitudinally. Wilson et al.^37^ found that the onset of irregular menses was most often within 3 months of EVD recovery and that over half of females had persistent irregular menses 10–12 months after ETU discharge. This suggests that there may be a critical period after illness when menstrual irregularities arise, although the time to resolution is variable. Although Godwin et al.^34^ found no significant association between pregnancy loss and time since ETU discharge, Fallah et al.^38^ found a much higher prevalence of stillbirths in pregnancies that occurred within 2 months of ETU discharge. PREVAIL III and de St Maurice et al.^35^ reported reduced prevalence of decreased libido over time, but Qureshi et al.^42^ found no significant difference in decreased libido in the acute convalescent (0–90 days) versus subacute convalescent (90–120 days) groups. Despite the reduction in decreased libido over time in PREVAIL III, the prevalence remained significantly higher in survivors than uninfected contacts at both visits. Previous studies conducted in Liberia and Guinea with 4 and 7 years of long-term follow-up data, respectively, found that most or all post-EVD sequelae decrease over time.^5^^,^^7^ However, neither study included reproductive health outcomes. It would be valuable for future studies to characterize the burden of adverse reproductive health outcomes across a longer period post-EVD.

### Limitations.

This systematic review has several limitations because of the design and biases within each included study. The lack of control groups in most studies makes it difficult to interpret whether prevalences of adverse reproductive outcomes are related to an overall higher prevalence in West Africa or to EVD. However, PREVAIL III did demonstrate a significantly higher prevalence of adverse reproductive health outcomes in survivors compared with uninfected contacts, suggesting that the postviral condition plays a unique role in women’s health. In addition, many of the studies took place at survivor clinics, which may be biased populations. Women may not have considered themselves ill enough to present to EVD survivor clinics, leading to an underestimated prevalence. Conversely, only the most ill survivors may report to the clinics, leading to an overestimated prevalence. Most studies only briefly investigated reproductive health as part of a comprehensive list of postillness sequelae. The studies largely rely on self-reported data, which introduce reporting and recall bias, and participants may have been reluctant to report their experiences to sensitive questions. Because the survey development process was not described, it is possible that other reproductive health outcomes are present in EVD survivors but were not included in surveys. Nonsignificant findings within the included studies may not have been reported in the manuscripts, leading to publication bias. Because our study often extracted data found only in the figures, tables, or supplemental materials and not in the main text or abstract, publication bias may be mitigated. Among cross-sectional studies, it is important to note that studies with a longer average time since ETU discharge may be associated with a higher prevalence of a particular outcome because of a longer period of exposure, and this is not necessarily reflective of later onset or persistence. Future studies should aim to address the limitations of the existing research to generate more robust evidence supporting the presence of adverse reproductive health outcomes in female EVD survivors.

Several studies were excluded from the meta-analysis because of the lack of extractable data, and there was significant between-study heterogeneity contributing to the underpowering of the meta-analysis. The subgroup analyses should be interpreted with caution: in particular, the decreased libido and pregnancy loss groups, which had fewer than five studies. It is also important to note that several studies contributed to multiple subgroups in the meta-analysis and that intratrial dependence was not accounted for. Moreover, studies with larger sample sizes carried greater weight regardless of quality. Future studies should provide sex-stratified granular data so that stronger meta-analyses can be performed. Because individual studies in this research area often use small cohorts, a highly powered meta-analysis would be useful in better understanding post-EVD reproductive health outcomes.

Finally, the studies identified in this systematic review were limited to the 2014–2016 West African EVD outbreak, likely because of its larger survivor population, although EVD outbreaks have also occurred in the Democratic Republic of the Congo (DRC), Sudan, Gabon, Uganda, and the Republic of Congo. The West African outbreak was the largest EVD outbreak in history, resulting in 28,610 cases and 17,302 survivors. In comparison, the largest outbreak outside of West Africa occurred in the DRC from 2018 to 2020, resulting in 3,470 cases and 1,183 survivors.^47^ A 2024 systematic review of general EVD sequelae throughout Africa described arthralgia, headache, myalgia, abdominal pain, fatigue, numbness of hands and feet, and hearing loss.^48^ Of the 23 studies identified, only 2 studies were conducted outside of West Africa (DRC and Uganda), neither of which investigated reproductive health outcomes.^49^^,^^50^ Studies are currently ongoing after the 2022–2023 Uganda outbreak, which may highlight sequelae experienced by these survivors.^51^

### Future directions.

This study highlights the burden of adverse reproductive health outcomes among female EVD survivors of the West African outbreak. Adverse reproductive health outcomes can be associated with osteoporosis, cardiovascular disease, anemia, mental health problems, and infertility, but these downstream impacts on physical and mental health have been infrequently evaluated.^52^ Future studies may choose to analyze associations between reproductive health and nonreproductive health outcomes to identify risk factors/predictive factors for adverse reproductive health outcomes or potential causal pathways. Providing sex-stratified data on nonreproductive health outcomes will also contribute to a better holistic understanding of post-EVD syndrome in women. Finally, the pathogenesis responsible for reproductive health outcomes in female EVD survivors is poorly understood, although the roles of stress, anxiety, weight loss, immune dysregulation, and persistent inflammation have been proposed.^34^^,^^38^ Despite the high prevalence of adverse reproductive health outcomes during acute LF infection, no studies were identified in our systematic review reporting reproductive health in LF survivors. Future studies may wish to investigate this topic to determine if the phenomenon is specific to EVD survivors or if it is found more generally in VHFs.

## CONCLUSION

Our systematic review and meta-analysis found that female EVD survivors experience significant impacts to their reproductive health after disease survival, including menstrual irregularities, pregnancy loss, and decreased libido. However, significant gaps exist in the current literature, and future research is needed to better characterize these outcomes and explore their long-term impacts. This knowledge will ensure that evidence-based public health interventions are enacted to improve the quality of life and overall health of female EVD survivors.

## Supplemental Materials

10.4269/ajtmh.23-0709 Supplemental Materials
