# Supplementary material for: Systematic Review and Meta-Analysis of Female Reproductive Health Following Ebola Virus Disease
Source: Am J Trop Med Hyg. 2024 Dec 17;112(3):663–73. doi: 10.4269/ajtmh.23-0709 (PMC11884292; doi:10.4269/ajtmh.23-0709)
Supplement: Supplemental Materials [file tpmd230709.SD1.pdf]

Supplemental Table 1: Search Strategy for Databases Included in Systematic Review

Database	Database Publisher/Vendor	Database Dates	Date of Last Search	Search Strategy
PubMed	NLM	1966-present	30-Nov-23	("Hemorrhagic Fever, Ebola"[Mesh] OR "Ebolavirus"[Mesh] OR "ebola"[tiab] OR "ebolavirus"[tiab] OR "Lassa Fever"[Mesh] OR "Lassa virus"[Mesh] OR "lassa fever"[tiab] OR "lassa virus"[tiab]) AND ("Survivors"[Mesh] OR "survivor*"[tiab] OR "Convalescence"[Mesh] OR "convalescence*"[tiab])
Ovid Medline	Ovid	1946-present	30-Nov-23	exp Hemorrhagic Fever, Ebola/ or exp ebolavirus/ or ebola.mp. or exp Lassa Fever/ or exp Lassa virus/ or lassa.mp. AND exp Survivors/ or survivor*.mp. or convalescence.mp. or exp Convalescence/
Embase	Elsevier	1947-present	30-Nov-23	('ebola hemorrhagic fever'/exp OR 'ebola hemorrhagic fever' OR 'ebolavirus'/exp OR 'ebolavirus' OR 'ebola' OR 'lassa virus'/exp OR 'lassa virus' OR 'lassa fever'/exp OR 'lassa fever') AND ('survivor'/exp OR 'survivor' OR 'convalescence'/exp OR convalescence)
Web of Science	Clarivate Analysis	1900-present	30-Nov-23	(ebola* OR lassa) AND (survivor* OR convalescence)
CINAHL Complete	EBSCO	1937-present	30-Nov-23	(ebola* OR lassa) AND (survivor* OR convalescence)
Global Health	Ovid	1973-present	30-Nov-23	(ebola* OR lassa) AND (survivor* or convalescence)

Supplemental Table 2: Power Analysis Calculations

	**Overall**	**Menstrual Irregularities**	**Pregnancy Loss**	**Decreased Libido**
p1	0.14			
p2	0.19			
**Effect size (*es*) using Cohen's *h**** **Pooled-Estimate Approach**	0.14			
**Effect size (*es*) using Cohen's *h***** **Heterogeneity Approach**	0.20	0.50	0.50	0.50
**Average sample size per group (*as*)**	196.62	165.23	79.67	288.33
**Number of effect sizes (*mk*)**	16	8	3	3
**Heterogeneity (*hg*)** †	3	3	3	3
**Power** **Pooled-Estimate Approach**	0.76			
**Power** **Heterogeneity Approach**	0.97	0.99	0.78	0.99

$$h = 2((\sin^{-1}\sqrt{p1}) - (\sin^{-1}\sqrt{p2}))$$
$$equation1 = \left(\frac{as + as}{(as)^2}\right) + \frac{(es^2)}{(2 \times (as + as))}$$
$$equation2 = hg \times equation1$$
$$equation3 = equation1 + equation2$$
$$equation4 = equation3/mk$$
$$equation5 = (es)/(\sqrt{equation4})$$
$$Power = (1 - \Phi(1.96 - equation5))$$

Post-hoc power calculation using pooled-estimate and heterogeneity approaches based on a two-tailed random effects model. The above equations were extracted from Valentine et al., and inputted into RStudio where calculations were performed.

*Effect size for the pooled-estimate approach was calculated based upon Cohen's *h* effect size formula.
**Effect size for the heterogeneity approach used pre-determined Cohen's *h* values: 0.2 for small and 0.5 for medium effect sizes.
†Heterogeneity determined as 0.33 for small, 1 for moderate, 3 for large. A large *hg* value was chosen due to significant heterogeneity.

Supplemental Table 3: Quality Assessment of Included Cross-Sectional and Observational Studies

	Research question	Study Population	Participation Rate	Subject Selection	Sample Size	Exposure Measured Prior to Outcome	Sufficient Timeframe	Different Levels of Exposure	Clearly defined exposure measures	Exposure measured over time	Clearly defined outcome measures	Assessors blinded	Low Loss to follow-up	Confounding variables	Score (out of 14)
De St Maurice	Yes	Yes	Yes	Yes	No	Yes	Yes	No	Yes	NA	Yes	No	NR	No	8
Fallah	Yes	Yes	NR	Yes	No	Yes	Yes	No	Yes	NA	Yes	No	Yes	No	8
Godwin	Yes	Yes	Yes	Yes	No	Yes	Yes	No	Yes	NA	Yes	No	NA	No	8
Guetiya Wadoum	Yes	Yes	Yes	Yes	No	Yes	Yes	No	Yes	NA	Yes	No	NA	No	8
Mattia	Yes	Yes	NR	Yes	No	Yes	Yes	No	Yes	NA	Yes	No	NA	Yes	8
Mohammed	Yes	Yes	Yes	Yes	No	Yes	Yes	No	Yes	NA	Yes	No	NA	Yes	9
Nanyonga	Yes	No	NR	Yes	No	Yes	Yes	No	Yes	NA	NR	No	NA	No	5
Qureshi	Yes	Yes	Yes	Yes	Yes	Yes	Yes	Yes	Yes	NA	Yes	No	NA	Yes	11
PREVAIL III	Yes	Yes	Yes	Yes	Yes	Yes	Yes	Yes	Yes	NA	Yes	No	Yes	Yes	12
Tiffany	Yes	Yes	Yes	Yes	No	Yes	Yes	Yes	Yes	NA	No	No	NA	Yes	9
Wilson	Yes	Yes	No	Yes	Yes	Yes	Yes	Yes	Yes	NA	Yes	No	NA	No	9
Wing	Yes	Yes	Yes	Yes	Yes	Yes	Yes	No	Yes	NA	Yes	No	No	Yes	10

NR = not reported; NA = not applicable to study; Quality assessment conducted by two independent reviewers (MD, CG) and discrepancies addressed by third reviewer (CZ).

Supplemental Table 4: Quality Assessment of Included Case Series

	Research Question	Study population		Consecutive Cases	Comparable Subjects	Intervention Description	Clearly defined outcome measurement	Follow-up Length	Statistical methods	Description of Results	Score out of 9
Kamali	Yes	Yes		NA	NA	Yes	NR	Yes	No	Yes	5

NR = not reported; NA = not applicable to study; Quality assessment conducted by two independent reviewers (MD, CG) and discrepancies addressed by third reviewer (CZ).
